# Feasibility of Temperature Control by Electrical Impedance Tomography in Hyperthermia

**DOI:** 10.3390/cancers13133297

**Published:** 2021-06-30

**Authors:** Redi Poni, Esra Neufeld, Myles Capstick, Stephan Bodis, Theodoros Samaras, Niels Kuster

**Affiliations:** 1 Department of Information Technology and Electrical Engineering, Swiss Federal Institute of Technology (ETH), 8092 Zurich, Switzerland; rponi@ethz.ch (R.P.); kuster@itis.swiss (N.K.); 2 Foundation for Research on Information Technologies in Society (IT’IS), 8004 Zurich, Switzerland; capstick@itis.swiss (M.C.); stephan.bodis@ksa.ch (S.B.); 3 Center of Radiation Oncology KSA-KSB, Kantonsspital Aarau, 5001 Aarau, Switzerland; 4 Department of Physics, Aristotle University of Thessaloniki, 54124 Thessaloniki, Greece; theosama@auth.gr

**Keywords:** perfusion estimation, temperature monitoring, conductivity reconstruction

## Abstract

**Simple Summary:**

Online treatment monitoring is an important tool to ensure the safety and effectiveness of hyperthermia cancer therapy. However, current solutions provide only sparse/inaccurate data, demand extensive access to complex and expensive infrastructure, or are associated with increased toxicity. In this study, we present a simulation-based evaluation of the feasibility of electrical impedance tomography (EIT) for hyperthermia treatment monitoring. EIT is a low cost, information-rich, non-invasive technique that could potentially be adapted and employed to reconstruct conductivity changes and translate them to temperature- and perfusion-change maps. Using an innovative reconstruction methodology that leverages (ideally personalized) treatment simulations, physics-motivated constraints, multiple frequencies, measurement-derived compensation, and novel numerical approaches, we investigated the impact of factors such as noise and reference model accuracy on the temperature- and perfusion-reconstruction accuracy. Results suggest that EIT can provide valuable real-time monitoring capabilities. As a next step, experimental confirmation under real-world conditions is needed to validate our results.

**Abstract:**

We present a simulation study investigating the feasibility of electrical impedance tomography (EIT) as a low cost, noninvasive technique for hyperthermia (HT) treatment monitoring and adaptation. Temperature rise in tissues leads to perfusion and tissue conductivity changes that can be reconstructed in 3D by EIT to noninvasively map temperature and perfusion. In this study, we developed reconstruction methods and investigated the achievable accuracy of EIT by simulating HT treatmentlike scenarios, using detailed anatomical models with heterogeneous conductivity distributions. The impact of the size and location of the heated region, the voltage measurement signal-to-noise ratio, and the reference model personalization and accuracy were studied. Results showed that by introducing an iterative reconstruction approach, combined with adaptive prior regions and tissue-dependent penalties, planning-based reference models, measurement-based reweighting, and physics-based constraints, it is possible to map conductivity-changes throughout the heated domain, with an accuracy of around 5% and cm-scale spatial resolution. An initial exploration of the use of multifrequency EIT to separate temperature and perfusion effects yielded promising results, indicating that temperature reconstruction accuracy can be in the order of 1 °C. Our results suggest that EIT can provide valuable real-time HT monitoring capabilities. Experimental confirmation in real-world conditions is the next step.

## 1. Introduction

Noninvasive imaging techniques such as electrical impedance tomography (EIT) are valuable tools for medical applications. EIT is used to image the electrical conductivity of tissues in the human body. EIT usage was first suggested in the 1970s [1]. Despite its relatively low cost, safety, and high temporal resolution, EIT has not been as widely adopted as other medical imaging methods, such as magnetic resonance imaging (MRI) and computed tomography (CT) [2,3].

The applications of functional EIT include pulmonary investigations [4], cardiac and gastrointestinal tract monitoring, breast cancer screening, and functional brain imaging [5]. Multiple devices have been introduced for clinical research [6], mainly in applications with functional imaging, such as bedside lung monitoring. Other applications, such as temperature estimation where accurate quantitative conductivity (or change in conductivity) reconstruction is needed, are more challenging compared with those in which only the volume with high dielectric change must be reconstructed.

One of the main challenges in EIT is that image reconstruction from measured voltages is an ill-posed problem [7]. Changes in the whole domain correspond to an infinite number of degrees of freedom (DOF) that must be reconstructed from a limited number of electrode measurements. Nevertheless, knowledge about distribution smoothness adds constraints, and regularization methods can be employed to facilitate reconstruction. Another issue with EIT is that impedances are affected by the entire volume rather than a single slice. Therefore, 2D reconstructions are merely an approximation of the real 3D problem [8]. While linearization methods combined with prior knowledge about the conductivity distribution and difference imaging have been used to further improve image reconstruction, the problem is nonlinear. Nonlinear reconstruction methods are more sensitive to inaccuracies in electrode models and positions. The literature on reconstruction algorithms for EIT [9] suggests that linear reconstruction methods should be combined with nonlinear iterative approaches to improve overall accuracy. From an instrumentation perspective, measurement noise, electrode positioning accuracy, signal generation, and sensing techniques impact overall EIT quality [10]. Further advances in EIT require improvements in both instrumentation and image reconstruction. Improvements are especially needed for applications that require the quantitative imaging of conductivity.

A potential application of EIT is in hyperthermic oncology. Hyperthermia (HT) therapy aims to selectively heat tumor tissue to temperatures ranging from 40 °C to 45 °C for a duration of about one hour. It is typically used as an adjuvant to radio- and/or chemotherapy in cancer treatment. In the case of deep-seated tumors, selective heating is usually achieved through coherent interference of electromagnetic (EM) energy from multiple radiating elements [11]. A significant challenge is noninvasive temperature monitoring in deep-seated tissue. The achieved temperature is difficult to predict, but it is important for tracking the achieved thermal dose in the tumor and avoiding potential treatment-limiting hotspots in healthy tissue. Treatment planning [12,13], which involves patient-specific EM simulation, optimization of energy deposition, and thermal prediction of the treatment, has been introduced as a tool for improving the prediction of thermal distribution. However, high uncertainty about the actual temperatures (e.g., due to perfusion changes during treatment) remains [14,15,16]. Research progress has been made in noninvasive monitoring using magnetic resonance thermometry (MRT) [17], but the accuracy of measurement is susceptible to patient movements, magnetic field drift over time, and limited sensitivity in fatty tissues, among others. In addition, the cost associated with MRT and the integration complexity with HT are high [18]. Alternatively, EIT can offer a low-cost, low-complexity solution for estimating temperature increases and perfusion changes during HT treatment.

Temperature elevation impacts tissue conductivity in two different ways. First, temperature changes the conductivity of intra- and extracellular fluids, which can be modeled by a linear relationship and described by a temperature coefficient ($T_{c}$)—the ratio of relative conductivity increases per degree centigrade. Second, in tissues in which thermoregulation-induced perfusion changes (e.g., vasodilatory response) are high, fluid flow in the extracellular environment increases, resulting in additional tissue conductivity change. At lower frequencies, current flows mainly in the extracellular region, whereas at higher frequencies, current flow is more uniform across all tissue compartments. Multifrequency EIT is considered as a method to distinguish changes in conductivity directly related to temperature (i.e., $T_{c}$-related part) from changes in conductivity due to perfusion increase [19]. Earlier studies have achieved temperature estimation accuracies ranging from 1.5 °C to 5 °C [20]. The results of these and other studies [21,22,23,24,25] suggest that to enable clinical EIT for HT and ablation treatment monitoring, improvements in conductivity reconstruction and temperature estimation are essential and consequently require accurate models of temperature-induced conductivity changes and the ability to distinguish temperature-related changes from tissue changes or damage-related changes, both permanent and temporary.

Recently, there has been increased interest and progress in applying EIT as a monitoring tool for thermal ablation, both in experimental and simulation studies [26,27,28,29], which motivates revisiting EIT for HT monitoring. New tools for EIT simulations have been introduced [30,31], and the computational power has increased considerably. Additionally, developments in tissue segmentation [32], combined with knowledge about tissue properties [33], enable the simulation of patient-specific treatment scenarios. Similarly, more realistic anatomical models can be used to perform sensitivity analyzes to improve the design of instruments. Most experimental studies in EIT are performed in tanks with simple geometrical shapes and few objects with different conductivities; hence, results cannot directly be translated to real human application. Since human anatomy is highly heterogeneous and geometrically complex, accurate representation in a model requires high resolution and many discretization elements. Notably, higher resolution negatively impacts reconstruction accuracy since total error minimization involves residuum minimization for more degrees of freedom, while the number of voltage electrode measurements remains the same [34].

Accurate temperature and/or perfusion estimation requires accurate knowledge about the relationship between temperature and conductivity, in addition to accurate conductivity imaging. In this paper, we focus on the achievable EIT reconstruction accuracy by using existing tools, such as electrical impedance tomography and diffuse optical tomography reconstruction (EIDORS) [31]), in conjunction with high-resolution anatomical models [35]. We aim to exploit HT treatment planning-based prior information and investigate the reconstruction of conductivity changes in the range expected for the given application. We also identify potential practical issues specific to hyperthermic oncology and their impact on the accuracy of conductivity change reconstruction to further improve temperature estimation.

## 2. Materials and Methods

To investigate the potential application of EIT in HT treatment planning and treatment monitoring, we performed simulations using the Virtual Population (ViP) Duke (age: 34, height: 1.77 m, BMI: 22.4 kg/m^2^) and Glenn (age: 84, height: 1.73 m, BMI: 20.4 kg/m^2^) anatomical models [35]. Two anatomical models were used to assess the impact of intersubject variability, as well as the importance of using personalized reference models. The models were discretized using a tetrahedral mesh in EIDORS v3.9. We first describe single iteration reconstruction using EIDORS. In Section 2.2, we present novel reconstruction approaches capable of overcoming the limitations of existing methods in our application of interest, their implementation, and the investigation scenarios. While considering the high heterogeneity of the human body, we then determined if the reconstruction accuracy improved when using a tissue-dependent penalty (TiD) parameter. A sensitivity analysis regarding the location and size of the simulated region was also performed.

In difference imaging, a reference model with an initial conductivity assignment is required. The measured changes in the electrode voltages are used to reconstruct the changes in conductivity from the reference model. As reference patient models may display anatomical segmentation inaccuracies, we investigated their impact on the reconstruction accuracy by considering a scenario where a volume outside the prior region, i.e., the volume, where an increased sensitivity is achieved by applying a penalty value [36], exhibited a large deviation (Δσ) from the reference model conductivity ($\sigma_{ref}$).

Noise in the measurement acquisition chain is also present in practical implementations. We assessed the impact of different electrode voltage noise levels (signal-to-noise ratio, SNR) on reconstruction accuracy using different reconstruction parameter values.

Finally, a realistic bladder tumor HT treatment scenario was considered as an EIT application case, using the two anatomical models (different body shapes and heating patterns). The clinical value of treatment planning-based EIT and the importance of personalizing the reference model were also assessed.

### 2.1. Single Iteration Reconstruction

EIDORS includes multiple algorithms for two- and three-dimensional (2D/3D) image reconstruction. In this study, we used difference imaging reconstruction on a 3D body. Single iteration reconstruction assumes small variations in conductivity, for which the relationship between voltage and conductivity can be approximated linearly as follows:

(1) y=Jx+n,

where $J_{ij}=\frac{\partial y_{i}}{\partial x_{j}}$ is the Jacobian, $x=\sigma -\sigma_{ref}$ is the difference of the actual conductivity distribution and the reference value, $y=v-v_{ref}$ is a vector with electrode voltage differences of the actual measurement to the reference measurement, and *n* is the measurement noise. Regularization techniques are used to solve this problem [36,37,38]. We used the one-step linear Gauss–Newton method to estimate $\hat{x}$ by minimizing the sum of quadratic norms for:

(2) $$||y-J\hat{x}{\left|\right|}^{2}+\lambda^{2}{\left||x|\right|}^{2}.$$

The solution of the above formulation is:

(3) $$\hat{x}={\left(J^{T}WJ+\lambda^{2}R\right)}^{-1}J^{T}Wy=By.$$

To reduce computational time by decreasing the size of the matrix to be inverted, *B* can be rewritten as follows:

(4) $$B=PJ^{T}{\left(JPJ^{T}+\lambda^{2}V\right)}^{-1},$$

where $P=R^{-1}$ and ${\left[R\right]}_{ii}={\left[J^{T}J\right]}_{ii}^{0.5}$. Effectively, *R* is a diagonal regularization matrix scaled with the sensitivity of each element and λ is a regularization parameter. $V=W^{-1}=I$ represents difference imaging EIT with identical channels. Two important and frequently used parameters in the reconstruction are the hyperparameter (λ) and the penalty parameter. When known changes are likely to occur in a smaller subdomain, a penalty parameter is used to implement an increased sensitivity in this region: ${\left[R\right]}_{ii}={\left[J^{T}J\right]}_{ii}^{0.5}{\left[Penalty\right]}_{ii}$, for *i* in the subdomain. More details about derivations can be found in existing publications [36,37,39].

### 2.2. Pipeline and Simulation Setup

The ViP Duke model comprised of tissue properties from the IT’IS tissue database [33] was imported into EIDORS. Only a 20 cm portion of the torso (620 k elements) was used for further analysis.

Using difference imaging, we exploited prior information about the geometry and the conductivity distribution. Hence, we focused on reconstructing the conductivity change ($\Delta \sigma_{rec}$):

(5) $$\Delta \sigma_{rec}=INV\left(v_{ref},v,\sigma_{ref}\right),$$

where reference conductivity (baseline for EIT difference reconstruction) is $\sigma_{ref}$. INV is the function to solve the inverse problem using *v* and $v_{ref}$ from the electrode voltages calculated from solving the forward problem or from current injection measurements for σ, respectively $\sigma_{ref}$. The actual conductivity change ($\Delta \sigma =\sigma -\sigma_{ref}$) is referred to as “modified conductivity”, since a range of conductivity change configurations will be created by modifying the reference conductivity to investigate difference image reconstruction scenarios.

HT treatment planning workflows already include a tissue segmentation step. The segmented anatomical model can be assigned tissue properties, while considering the EIT frequency, to establish the reference model.

Difference imaging reconstruction is less sensitive to the modeling of the electrodes and contact impedance, as we assume the same conditions are present in both reference and additional measurements of the model to be reconstructed [9,40]. Therefore, we did not investigate the impact of the electrode parameters in this study; however, changes in the contact quality in experimental measurements will affect the reconstruction accuracy.

**Figure 1:**
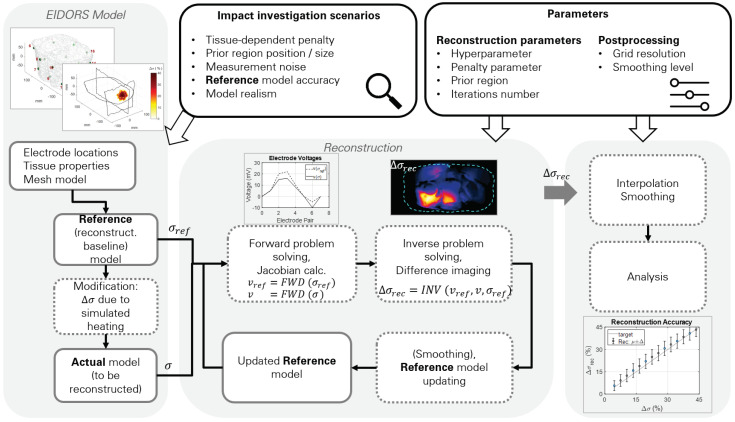
Illustration of the implemented reconstruction pipeline and the scenarios investigated in this study. Boxes with continuous outlines represent data, while the dotted ones represent processes. First, the actual and the reference model are generated, based on a discretized dielectric model of the patient and electrodes. Reconstruction proceeds through multiple iterations of forward (FWD) and inverse (INV) problem solving. The reconstruction results have been analyzed to study the impact of reconstruction approaches, noise, as well as reference model realism and accuracy.

**Figure 2:**
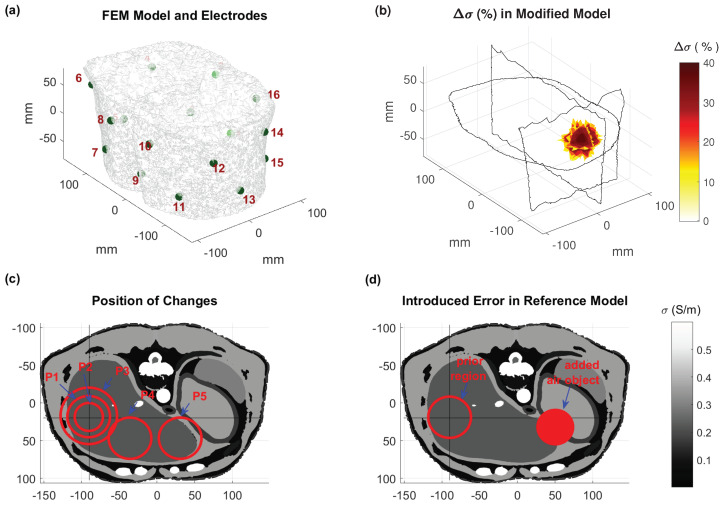
(**a**) FEM model of the Duke anatomical model torso with electrode locations indicated in green; (**b**) slices of the modified model Δσ(%) for a heated region in the liver; (**c**) locations and sizes of the different heated region scenarios; (**d**) setup featuring changes outside the prior region.

The reconstruction pipeline used in this study is shown in Figure 1. The reference model includes the tissue conductivity assignments. In nonablative HT treatment, the target region can reach temperatures of up to 45 °C. In addition to the direct temperature-related conductivity change contribution modeled with temperature coefficients (∼2%/°C), σ can also change due to perfusion changes, thus altering the tissue extracellular fluid distribution. From the reference model, modified models were created by changing the tissue conductivity by up to 40%, a level similar to the experimental measurement study by Gersing [25]. The reference conductivity was multiplied by a 3D Gaussian shape mimicking heating during an HT treatment, as shown in Figure 2. At the end of the simulation pipeline, we compared the reconstructed model conductivity with the modified model conductivity.

For current injection and measurement, we positioned electrodes in single nodes, distributed as two rows of eight electrodes to form an interleaved arrangement. Current injection was applied transversely through single pairs (1–8, 2–9, etc.) and the voltage difference was calculated in all adjacent pairs ($v_{34}$, $v_{45}$, etc.) except for the electrodes used for current injection. Injecting currents through transversal, nonadjacent electrodes increases the current density, and hence, the sensitivity of EIT to changes in deeper tissues. Theoretically, a larger number of electrodes should improve the reconstruction accuracy. However, for the same injected current, which is limited by safety considerations, the voltage difference of more densely placed electrodes will be lower and, in practice, we will obtain more voltage measurements with lower SNR. An additional drawback of using numerous electrodes is the increased computational reconstruction effort. The torso model and electrode placements used in this study are shown in Figure 2.

In HT applications, the prior region can be determined either from the volume with highest HT power deposition or from a preliminary thermal simulation, which does not have to exactly reproduce the real patient tissue parameters. Although prior regions improve reconstruction by focusing on changes in a smaller volume, changes outside the prior region may be attributed to changes inside the region.

Simulations were interpolated to a structured rectilinear grid. A spatial averaging filter (cubic volume of 1.2 cm edge length) was applied to Δσ (%), mimicking the expected smoothness of the heat distribution in tissue. Finally, the reconstruction accuracy across different tissues was analyzed.

### 2.3. Investigation Scenarios

#### 2.3.1. Tissue-Dependent Penalty

As the human body is highly heterogeneous, a wide range of low frequency σ values can be found, from close to 0 S/m (internal air) to over 0.36 S/m (muscle) and 3 S/m (urine). The prior region can encompass a multitude of tissues covering a broad σ range, which makes adequate change detection throughout the entire range without overestimation or underestimation challenging. Information from the reference model $\sigma_{ref}$ allows for the use of tissue-dependent penalty values, as constant penalty changes in low σ tissues are overestimated and changes in tissues with high σ, such as muscle, are underestimated, since the reconstruction algorithm minimizes the overall electrode voltage differences. In this study, we compared the reconstruction accuracy in different tissues when using a constant penalty value versus a tissue-dependent penalty in a single iteration reconstruction without applying any averaging or smoothing filter.

#### 2.3.2. Region Location and Size

It is necessary to compare the reconstruction accuracy for different heating locations and sizes. The size of the region where the conductivity was changed should correspond to the typical extent of focused heating in HT treatment. For the first investigated location, spherical heating regions of different diameters were considered. A simulated heating was applied to a spherical region, as illustrated in Figure 2, by multiplying the conductivity inside the sphere with $1+0.4\cdot e^{-\frac{r^{2}}{2R^{2}}}$ (*r*: radial distance, R=3 cm, peak σ-increase of 40%). Depending on the location, the region can contain more than one tissue type. The modified regions are shown in Table 1 and illustrated in Figure 2. In these scenarios, we assumed that the prior region is perfectly known, and corresponds to the heated region.

#### 2.3.3. Impact of Inaccurate Reference Model

The reconstruction is sensitive to the accuracy of the reference model. Even if the reference model is accurate at the beginning of the treatment, organ shifts and air movement in the bowels can occur during the treatment, since the HT treatment duration is relatively long. We simulated such a scenario, where in addition to the changes due to the simulated heating, the modified model included a spherical region with a 50 mm diameter and σ = 0 S/m (same as air), as illustrated in Figure 2. The setup corresponds to P1 (Table 1), such that results can be compared with the ideal case of an accurate reference model (see Section 2.4 and Section 3.3.1 regarding the impact of not using a personalized reference anatomy).

In addition to the iterative approach with a fixed prior region, we also introduced an adaptive prior region approach. An initial mask was obtained by reconstructing the conductivity change without prior region and thresholding locations, where a high change was obtained. Subsequently, the mask was used as a prior region and a relatively relaxed penalty value of 0.1 was assigned before obtaining an adapted or more focal mask. On the basis of the obtained reconstruction, three additional reconstruction iterations with a stricter penalty value were performed in the usual manner. This adaptive approach increased the reconstruction sensitivity to changes outside of the classic prior region. Both the change in the simulated heated region and the unexpected change outside the prior region were simultaneously reconstructed. To achieve this, five (1 + 1 + 3) iterations instead of three were required.

#### 2.3.4. Voltage Measurement Noise

The reconstruction problem is ill-conditioned. Minor voltage differences in the electrode measurements can lead to large changes in estimated conductivity. As a result, electrode voltage measurement noise is expected to significantly corrupt the reconstruction quality. Here, we investigated its impact on the conductivity reconstruction in the Duke anatomical model with a large number of mesh elements. Specifically, we assessed the impact of the noise level by adding noise with different SNR levels in setup P1. A noisy voltage vector ($v_{n}$) was generated by adding noise to the electrode voltages (*v*) from the forward problem solution of the modified model (σ).

(6) $$v_{n}=v+n,$$

where *n* is zero mean white Gaussian noise with standard deviation $\sigma_{n}$. The SNR is calculated as follows:

(7) $$SNR=20\log\left(\frac{\Delta v_{rms}}{\sigma_{n}}\right),$$

where $\Delta v=v-v_{ref}$.

### 2.4. Simulated HT Treatment Reconstruction

**Figure 3:**
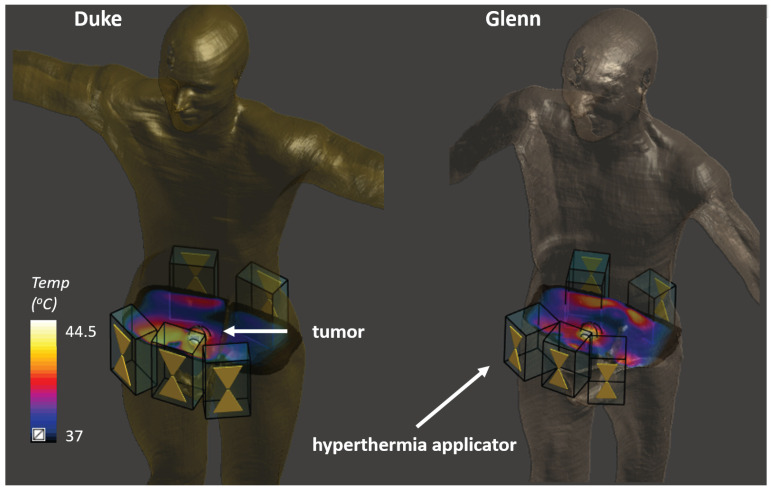
Simulated HT treatment in the Duke and Glenn anatomical models. Five modular applicator elements were placed circumferentially around the tumor, and their phases and amplitudes were optimized to preferentially heat the tumor. Two different anatomical models were used to investigate the impact of anatomical variability, as well as the impact of using a nonpersonalized reference model for reconstruction.

In this part of the study, we used a setup that mimicked the targeting of a bladder tumor using locoregional HT, where heat was delivered to a larger region encompassing the tumor (see Figure 3). This scenario provides increased realism and avoids the simplifying symmetries of the previous sections.

**Figure 4:**
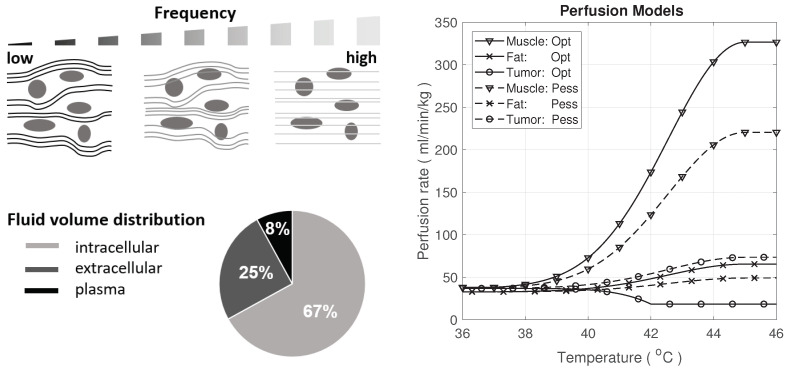
Current flow at different frequencies and fluid distribution in the human body [42] (**left**); optimistic and pessimistic perfusion models for muscle, fat, and tumor tissue [14,43] (**right**).

The procedure for the simulation was as follows:

1. We performed two thermal simulations of a one-hour treatment ($T_{Opt}$ and $T_{Pess}$) using the same specific absorption rate (SAR) distribution. The Pennes bioheat equation (PBE) [41] with temperature-dependent perfusion models was used for the thermal simulations (see Equation (8)). The applied power level was the same in both cases, but the temperature-dependent perfusion models for muscle, fat, and tumor tissues were different (see Figure 4), to illustrate the impact of perfusion uncertainty;
2. We translated the temperature increase to a modified conductivity map, which included a component directly related to temperature ($\Delta \sigma_{temp}$) and a perfusion-related indirect component ($\Delta \sigma_{perf}$);
3. We reconstructed and analyzed the changes in conductivity based on the “ground truth” temperature simulation ($T_{Pess}$), using the conductivity at 37 °C as the reference model (Scenario 1) or the conductivity for the “planned” $T_{Opt}$ (Scenario 2). The reconstructed conductivity was then converted into a reconstructed temperature estimation map.

**Figure 5:**
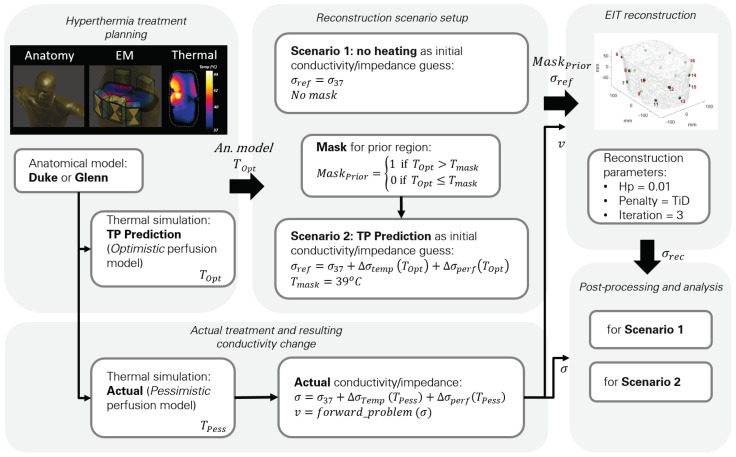
Conductivity changes reconstruction pipeline for two investigated EIT scenarios: EIT attempts to reproduce the voltage measurement signal of the “actual” model by reconstructing temperature and perfusion changes with regard to the reconstruction reference. (Scenario 1) uses the conductivity at 37 °C as reconstruction reference, while (Scenario 2) uses the modified conductivity as predicted by computational modeling of induced heating, perfusion response, and resulting conductivity change (but wrongly assuming an “Optimistic” perfusion, while the “actual” conductivity change is based on the “Pessimistic” perfusion model). Scenario 2 also employs masking based on the predicted temperature increase (prior region) to improve reconstruction.

The procedure is illustrated in Figure 5.

The PBE couples thermal diffusion with a heat-sink term that is proportional to the local perfusion and to the difference between the local tissue temperature (T(t)) at time *t* and the arterial blood temperature ($T_{a}$):

(8) $$\rho c\frac{\partial T}{\partial t}=\nabla k\nabla T+w_{b}\rho_{b}c_{b}\left(T_{a}-T\right)+q_{m}+q_{ext},$$

where ρ represents density (kg/m^3^), *c* is the specific heat capacity (J/kg°C), *k* is the thermal conductivity (J/(s·m·°C)), $w_{b}$ is the perfusion rate (kg/(s·m^3^)), $\rho_{b}$ is the density of blood, $c_{b}$ is the specific heat capacity of blood, $q_{m}$ is the metabolic heat generation rate (J/(s·m^3^)), and $q_{ext}$ is the electromagnetic power deposition. $w_{b}$ can be temperature dependent to account for vasodilation.

#### 2.4.1. Change in Conductivity Due to Temperature Increase

The change in conductivity during a HT treatment can be modeled with two components:

(9) $$\Delta \sigma \left(T\right)=\Delta \sigma_{temp}\left(T\right)+\Delta \sigma_{perf}\left(T\right),$$

where the change in conductivity directly due to the increase in temperature ($\Delta T=T-T_{ref}$) is $\Delta \sigma_{temp}\left(T\right)=T_{c}\cdot \Delta T$, with the temperature coefficient $T_{c}$ (we assume $T_{c}$ = 2%/°C in all tissues).

The conductivity change due to perfusion depends on the tissue as well as the frequency. At lower frequencies (kHz, LF), current flows mainly in the extracellular compartment, whereas at higher frequencies (MHz, HF), current flow tends to be more uniform across all tissue (see Figure 4).

For the purpose of this study, a simple model representing the difference between high and low frequency EIT and perfusion effects was constructed. Large uncertainties were associated with the temperature dependence of the perfusion $\Delta \sigma_{perf}$. However, as long as the model reproduced the general magnitude and behavior in terms of perfusion impact on conductivity and heating, it did not affect the generality of the study conclusions. To model the different frequencies, we neglected other dispersive effects and assumed that:

(10) $$G_{HF}=G_{icHF}+G_{ecHF}+G_{pHF},$$

and

(11) $$G_{LF}=G_{ecLF}+G_{pLF},$$

where $G_{LF}$ and $G_{HF}$ are the LF and HF conductance, respectively. $G_{ic}$, $G_{ec}$, $G_{p}$, correspond to intracellular, extracellular (without the blood plasma), and plasma conductance. We used the average human body volume ratio of these compartments from [42], as illustrated in Figure 4, as the conductance ratio between compartments to model the perfusion impact on conductance. Actual values are tissue-dependent.

The change in perfusion affects the total conductance by changing the relative contributions of the three compartments; therefore, the plasma volume increases as the perfusion increases. Assuming that the relative tissue volume change related to a perfusion increase is small and the plasma conductivity is the principal contributor to the overall conductivity, we obtain:

(12) $$\Delta \sigma_{perf}\left(T\right)=\frac{\Delta V_{p}\left(T\right)}{V_{total}}\left(1+T_{c}\cdot \Delta T\right),$$

where $\Delta V_{p}\left(T\right)=V_{p,0}\cdot \left(\sqrt{\omega \left(T\right)}-1\right)$ is the plasma volume change due to perfusion, and $\omega \left(T\right)$ is the relative perfusion change. $\alpha =\frac{V_{p,0}}{V_{total}}$ is the relative amount of plasma prior to heating, which is taken uniformly as 8% (see Figure 4), while in reality it varies across tissues and individuals. The −1 accounts for the plasma volume prior to heating, which is already included in the $\Delta \sigma_{temp}$ term. The square root approximates the relationship between blood vessel cross-sectional area and perfusion increase and is obtained under the assumption of a constant pressure drive and laminar flow [44].

Perfusion-related changes were considered for muscle, fat, and tumor tissues, particularly for prominent tissues with strong temperature dependence of perfusion and an important impact on the predicted temperature, using the two perfusion models shown in Figure 4, according to [14]. Tumor perfusion has a high associated uncertainty [14] due to irregular vascularization.

##### Disentangling Temperature and Perfusion

Distinguishing $\Delta \sigma_{temp}$ from $\Delta \sigma_{perf}$ to identify temperature and perfusion changes is not the subject of this paper. However, a possible approach is provided here:

$\Delta \sigma =\Delta \sigma_{temp}+\Delta \sigma_{perf}$, where $\Delta \sigma_{temp}=T_{c}\cdot \Delta T$ and $\Delta \sigma_{perf}=\alpha \cdot \left(\sqrt{\omega }-1\right)\cdot \left(1+T_{c}\cdot \Delta T\right)$. If α is known at two frequencies for a tissue of interest (in this study, we assumed $\alpha_{LF}$ = 24% and $\alpha_{HF}$ = 8% for all tissues), we obtain

(13) $$\Delta \sigma_{LF}-\frac{\alpha_{LF}}{\alpha_{HF}}\cdot \Delta \sigma_{HF}=\left(1-\frac{\alpha_{LF}}{\alpha_{HF}}\right)\cdot T_{c}\cdot \Delta T$$

and

(14) $$\Delta \sigma_{LF}-\Delta \sigma_{HF}=\left(\alpha_{LF}-\alpha_{HF}\right)\cdot \left(\sqrt{\omega }-1\right)\cdot \left(1+T_{c}\cdot \Delta T\right).$$

The former can be used to estimate ΔT, while the latter can be used to obtain ω (either using the ΔT estimated using Equation (13), or ΔT from the simulation, or neglecting the term $T_{c}\cdot \Delta T$ in Equation (14)). In practice, $\alpha_{LF}$ and $\alpha_{HF}$ might not be known, as the exact form of the temperature and perfusion dependences likely deviates from Equation (12), and the reconstructed $\Delta \sigma_{LF}$ and $\Delta \sigma_{HF}$ contain reconstruction errors. For a brief analysis of the latter, see Section 3.3.2.

#### 2.4.2. Reconstruction Scenarios

Temperature predictions have uncertainties; hence, the need for online monitoring of temperature during treatment. Here, we assumed that a temperature distribution ($T_{Pess}$) corresponds to the actual thermal treatment administered to a patient with a bladder tumor. Using the equations and assumptions above, we calculated the actual conductivity change corresponding to $T_{Pess}$ and the corresponding EIT voltages were used for reconstruction. Two scenarios were considered: one without previous knowledge and one with an imperfect thermal simulation-based treatment plan. In Scenario 1 (see Figure 5), we reconstructed conductivity changes using the values at 37 °C (no heating applied) as the reference conductivity. In Scenario 2, temperature distributions from simulations using $T_{Opt}$ were used to define the prior region for reconstruction; the incorrect perfusion information was used to introduce uncertainty similar to expected outcomes in a real treatment. Despite not using accurate perfusion values, Scenario 2 provided a better starting point than Scenario 1 for the reconstruction, as the conductivity difference to be reconstructed is smaller.

In both scenarios, we determined the prior region by thresholding $T_{Opt}$ at a temperature above a temperature threshold ($T_{Mask}$). For Scenario 2, a $T_{Mask}$ of 39 °C was used, whereas for Scenario 1, no mask is applied. In Scenario 1, we expected changes in the whole volume, whereas in Scenario 2, the prior region was smaller, as differences were more localized. Suitable temperature thresholds were identified by studying the resulting reconstruction accuracy in a range of setups. Higher thresholds prevent the reconstruction outside the masked volume, resulting in an overall increased error, while lower thresholds lead to underestimation of tumor heating, as the impedance changes are attributed to a larger region.

To investigate the importance of using personalized reference models, reconstruction was performed again using the Duke reference model, but with a simulated HT treatment measurement of Glenn (similar element placement, same steering parameters). As reconstruction with 16 elements was unsuccessful (see Section 3.3.1), eight-element reconstruction was investigated further. Subsequently, changes in anatomy (Glenn has a smaller cross-section area than Duke) were compensated by rescaling the voltages with the ratio of the voltages prior to heating. The additional constraint of demanding a positive temperature increase was imposed by zeroing all negative conductivity changes prior to each reconstruction iteration (note: negative conductivity changes cannot be excluded completely, e.g., due to geometry changes during treatment or perfusion redistribution; performing the reconstruction step after zeroing does allow to account for some of that). Finally, the expected temperature distribution smoothness was mimicked by convolution with a Gaussian filter (radius: 1 cm; chosen based on the characteristic lengths of the PBE Green’s function in muscle, bone, fat, and tumor at the initial temperature).

For the analysis of the reconstruction accuracy, the conductivity change in the heated reference model was compared with the conductivity change in the reconstruction.

Both the LF and the HF EIT cases were simulated. In the LF case, the contribution of perfusion to the conductivity change is higher. Thus, the same temperature distribution resulted in a higher total change in conductivity. Ultimately, EIT at multiple frequencies was used to distinguish conductivity changes related to $T_{c}$ from indirect conductivity changes related to perfusion changes and to monitor both the temperature and the perfusion distribution. In this study, we focused on the feasibility of conductivity reconstruction, and only briefly considered multifrequency EIT-enabled contribution separation.

## 3. Results

### 3.1. Reconstruction Time

A typical reconstruction for a setup with ∼6E5 tetrahedral elements, 16 electrodes, and 182 voltage measurements requires less than 5 min on a personal computer with an Intel i7-4770 processor (3.4 GHz, 4 cores). If less than three iterations are used, the reconstruction speed can be further accelerated. In view of the characteristic heating time in hyperthermic oncology, this provides sufficient temporal resolution.

### 3.2. Investigation Scenarios

#### 3.2.1. Tissue-Dependent Penalty

**Figure 6:**
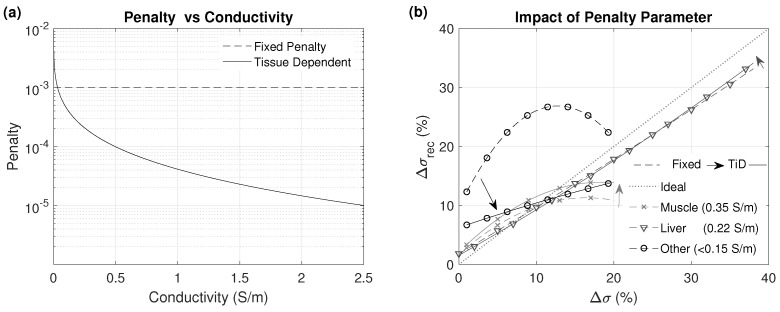
(**a**) “Tissue-dependent Penalty” and “Fixed Penalty” values. (**b**) Plot by tissue of the fitted relationship between reconstructed ($\Delta \sigma_{rec}$) versus reference (Δσ) changes in conductivity using “Fixed Penalty” (dashed line) and “Tissue-dependent Penalty” (solid line).

Second order polynomials were fitted to the scatter plots of actual conductivity change ($\Delta \sigma =\sigma -\sigma_{ref}$; in %, relative to nonheated baseline conductivity) vs. reconstructed conductivity ($\Delta \sigma_{rec}=\sigma_{rec}-\sigma_{ref}$). Figure 6 shows the results from a single iteration without any averaging filter after the reconstruction. The fits were performed separately for all tissues present in the heated regions. Results showed that a constant penalty value applied to all the tissues leads to an overestimation for low σ tissues and an underestimation in the reconstruction of the high σ tissues. Tissue-dependent penalty values improve the reconstruction across all tissues.

#### 3.2.2. Multiple Regions

**Figure 7:**
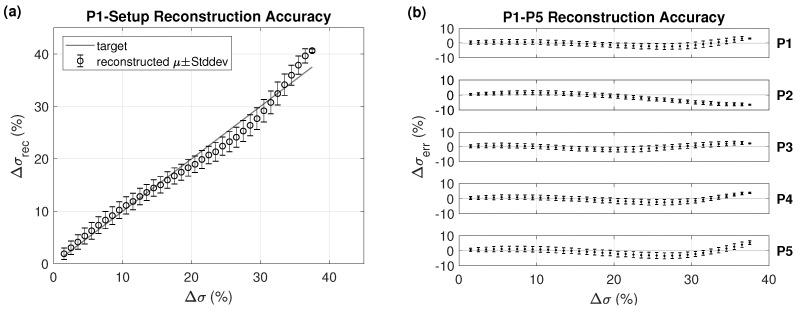
(**a**) Reconstructed conductivity $\Delta \sigma_{rec}$ (%) for the P1 setup from Figure 2 and (**b**) its deviation from the actual conductivity change ($\Delta \sigma_{err}=\Delta \sigma_{rec}-\Delta \sigma$) for all the setups P1–P5, as shown in Table 1 and illustrated in Figure 2, (right), using three iterations, tissue-dependent (TiD) penalty, and Hp = 0.01.

For scenario P1 from Table 1, Figure 7 shows the $\Delta \sigma_{rec}$ (%) versus Δσ (%), mean, and ± standard deviation after computing a sliding histogram every 1% of Δσ. The results for all other cases are shown as the error in conductivity reconstruction ($\Delta \sigma_{err}=\Delta \sigma_{rec}-\Delta \sigma$ in %) from the modified Δσ (%). The magnitude of the deviations of the reconstructed conductivity changes from the actual changes, as well as their variability, are similar and moderate (well below 10%) in all cases. The largest deviation from the target is observed in the case of smaller region (P2), which corresponds to the situation with the highest dielectric contrast at the heating region surface.

#### 3.2.3. Impact of Inaccurate Reference Model

**Figure 8:**
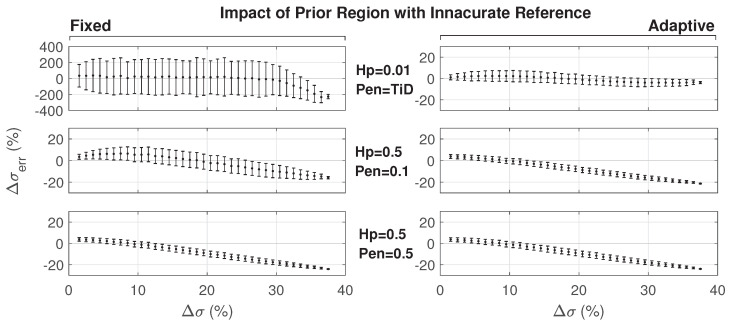
Reconstructed deviation in an inaccurate reference model (large inserted air sphere) for a fixed prior region using three iterations (**left**) and an adaptive prior region using 1 + 1 + 3 iterations (**right**)—note the different scale in the upper left graph.

When investigating the impact of changes outside the prior region, which is also equivalent to an inaccurate reference model conductivity, both the penalty parameter and the hyperparameter values are important. The hyperparameter shows the reliance of the reconstructed model on the reference model σ. The penalty, however, impacts how much the reconstruction focuses on changes within the prior region and how much it relies on the absence of no changes outside of the prior region. Since both of these parameters are related to the accuracy of the reference model, results for different values of these parameters are presented in Figure 8.

Initially, we observed that the reconstruction error resulting from the important conductivity changes outside the prior region can be large when using the same penalty and hyperparameter values, such as for the ideal case. Relaxing those parameters reduced the standard deviation of the error, but the reconstructed conductivity still did not follow the expected change across the whole range. After introducing adaptive prior regions, an improvement of the results was obtained, as shown for TiD penalties in Figure 8. Refer to Section 3.3.1 regarding reconstruction using a nonpersonalized reference anatomical model.

#### 3.2.4. Voltage Measurement Noise

**Figure 9:**
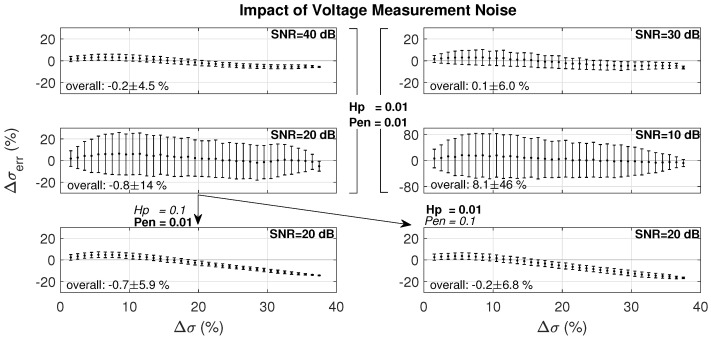
Impact of electrode voltage SNR (see Section 2.3.4 for the SNR calculation) on the reconstruction accuracy using three iterations for four levels of SNR (10, 20, 30, 40 dB), and in the 20 dB SNR case for varying combinations of reconstruction parameters (hyperparameter and penalty)—note the different scale in the 10 dB SNR case.

Figure 9 shows the results for heating scenario P1, when voltage measurements with different levels of SNR are mimicked. Since we wanted to focus on the impact of noise, the prior region was fixed and there were no changes outside the prior region in the reference model. The SNR was calculated with respect to the voltage difference, not the absolute voltages. As expected, noise strongly deteriorated the reconstruction accuracy, especially with low hyperparameter values. In the reconstruction algorithm, this gives more weight to the voltage measurements, which have poor SNR in this case, and less to the reference conductivity in the regularization part.

### 3.3. Simulated HT Treatment Reconstruction

**Figure 10:**
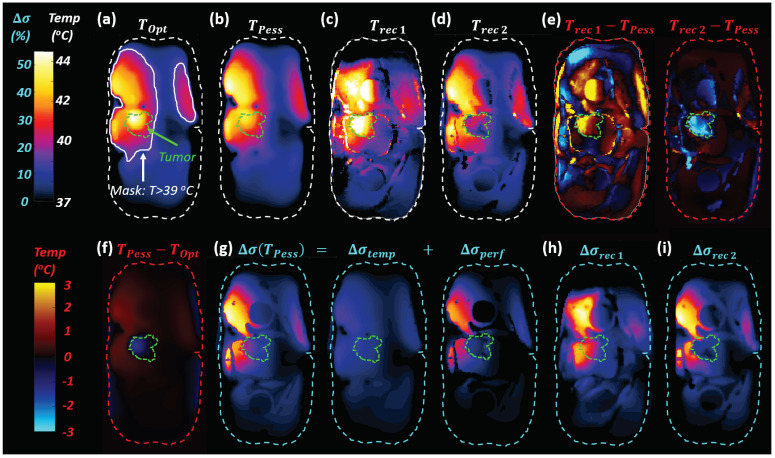
Reconstruction results from realistic HT treatment modeling (LF, Duke anatomical model): (**a**) axial slice from the thermal simulation with the optimistic perfusion model ($T_{Opt}$), (**b**) axial slice from thermal simulation with the pessimistic perfusion model ($T_{Pess}$) and (**f**) difference between $T_{Pess}$ and $T_{Opt}$; (**c**,**d**) reconstructed temperature results from Scenario 1 ($T_{rec\;1}$, using the reference conductivity at 37 °C) and Scenario 2 ($T_{rec\;2}$, using the reference conductivity $\Delta \sigma (T_{Opt})$), as calculated from the reconstructed $\Delta \sigma_{rec}$ in (**h**,**i**), respectively; (**e**) temperature estimation error for both scenarios; (**g**) $\Delta \sigma (T_{Pess})$ with its direct temperature-related ($\Delta \sigma_{temp}$) and the perfusion-related $\Delta \sigma_{perf}$ contributions.

**Figure 11:**
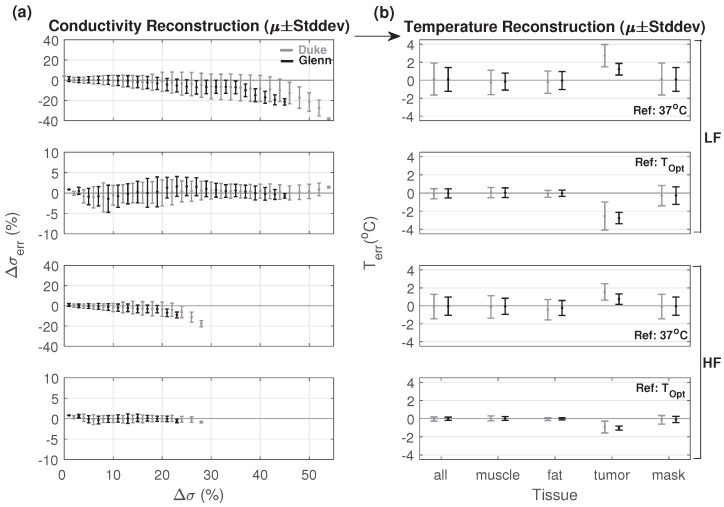
(**a**) Reconstructed mean and standard deviation of conductivity ($\Delta \sigma_{err}$) and (**b**) estimated temperature error ($T_{err}$). Reconstructions were performed for the Duke and Glenn anatomical models, for low frequency (LF) and high frequency (HF) current injection, using the conductivity map at 37 °C as the reference (baseline for EIT difference reconstruction) or the one predicted by thermal simulations with the (inaccurate) optimistic perfusion model ($T_{Opt}$). Mean and standard deviation of temperature error for muscle, fat, tumor, the prior region mask, and all tissues combined are shown. TiD penalty and Hp = 0.01 were used.

Slice views of simulated HT treatment temperatures, conductivity maps, and reconstructions are shown for Scenario 1 and 2 (Duke model, LF EIT) in Figure 10. Simulations were performed using the Duke and Glenn anatomical models for LF and HF EIT, using reconstruction Scenarios 1 and 2, resulting in a total of eight cases. For all these cases, we also calculated the estimated temperature change error ($T_{err}=T_{rec}-T_{actual}$) for fat, muscle, tumor, and all tissues combined by using the reconstructed conductivity (see Figure 11). $T_{rec}$ was calculated using the inverse of the function Δσ(T) that was used to translate the temperature distribution to a modified conductivity map (see Section 2.3). This only served to provide an interpretable error metric for analysis purposes. In practical applications, the uncertainties associated with the temperature dependence of conductivity prevented reliable inversion and multifrequency EIT measurements instead permitted direct temperature reconstruction by separating direct temperature-related from perfusion-related conductivity changes (see Section 3.3.2).

In Scenario 1, there was higher error across the whole range of change, as a result of the much larger conductivity changes (up to 55%) and the large required prior region. In Scenario 2, we observed an improved reconstruction accuracy, except for the tumor. Even though there were relatively high changes in tumor conductivity (up to 20%), tumor volume was relatively small and the reconstruction attributed the associated impedance changes to small conductivity changes in a larger volume. As a result, changes in tumor conductivity were only partially reconstructed for Scenario 2.

HF reconstruction resulted in lower temperature prediction errors compared to the LF case. This was mainly due to the smaller range of the conductivity change that required reconstruction.

Similar observations were made for the Duke and Glenn anatomical models, with slightly better results for Glenn, perhaps due to his smaller body size. The heating pattern in Glenn was more focused compared to Duke, resulting in a smaller region of significant conductivity changes and thus a smaller prior region, facilitating reconstruction.

For the simulated HT treatment on the Duke model, Table 2 shows the electrode voltage levels obtained by solving the forward problem when injecting a 1 mA current at the stimulating electrodes. The injection current was limited by safety constraints. For frequencies above 100 kHz, the stimulating current should not exceed 10 mA regardless of the pulse shape. In a practical implementation, depending on the SNR and the acquisition system capabilities, the conductivity-change-induced reconstruction-relevant voltage differences can be of a similar magnitude as the noise.

The results presented in Table 2 also show that in Scenario 2 the remaining difference between the reference and reconstruction-based voltage in the second and third iteration was much smaller than for Scenario 1. This was a consequence of using $T_{Opt}$ as a starting guess, which was closer than Scenario 1 to the target distribution. In fact, in Scenario 2, the first iteration would be sufficient. Nevertheless, three iterations were maintained, in case of important conductivity changes within or outside the prior region, which cannot be excluded in advance. The expected electrode voltages and voltage changes are important for defining the hardware requirements.

#### 3.3.1. Personalized Reference Model

**Figure 12:**
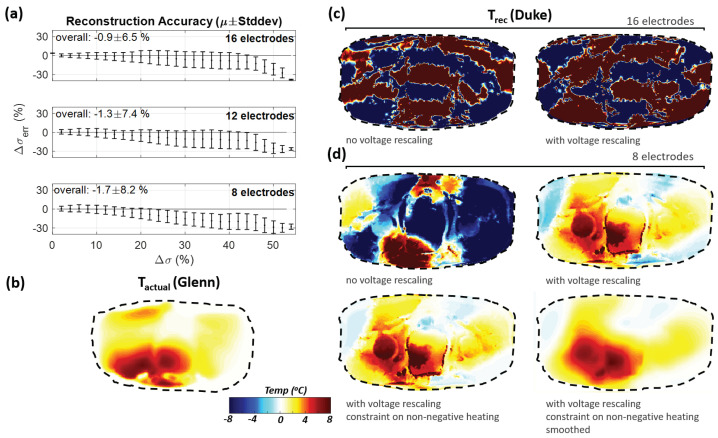
(**a**) Conductivity reconstruction error ($\Delta \sigma_{err}=\Delta \sigma_{rec}-\Delta \sigma$ in %) using 16, 12, or 8 electrodes, when the actual treatment (along with the extraction of the measurement voltages) is applied to the Duke model, (**b**) actual heating on Glenn, (**c**,**d**) reconstruction is performed using the Duke model as reconstruction reference, to study nonpersonalized reconstruction of heating on Glenn. While avoiding the generation of patient-specific models for reconstruction considerably reduces the involved effort, an important factor in a clinical environments, it also results in reduced reconstruction accuracy. As hyperthermia QA guidelines recommend personalized treatment planning for deep-seated tumors, personalized models are frequently available already. The important reconstruction errors in (**a**) reflect the use of the Duke conductivity distribution at 37 °C as reconstruction reference, while the reconstruction approaches in (**c**,**d**) employ nonpersonalized, Duke-based treatment planning (incl. thermal modeling) instead. (**c**) displays reconstruction results obtained using 16 electrodes with or without voltage-rescaling to compensate for the absence of a personalized reference model. (**d**) displays reconstruction results obtained when reducing the number of electrodes to 8, using voltage-rescaling, introducing constraints (non-negative temperature changes), and applying Green’s-function-based smoothing. These measures result in increasingly accurate temperature increase estimations.

**Figure 13:**
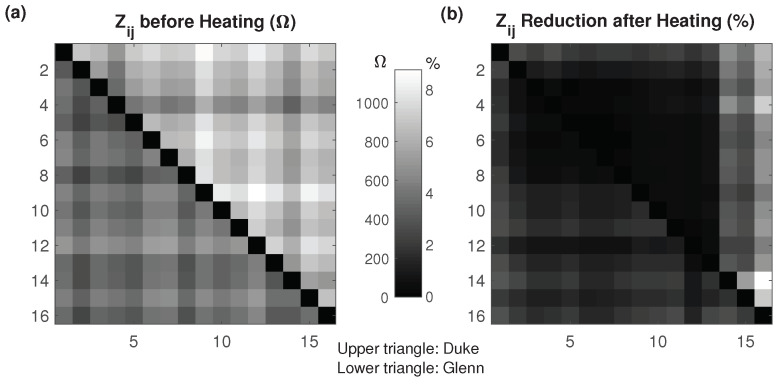
(**a**) Preheating impedance $Z_{ij}$ per electrode pair (in Ω) and (**b**) impedance reduction due to heating (in %). Upper and lower triangle values correspond to the Duke and Glenn anatomical models, respectively. The numbering follows Figure 2.

When the voltages from a simulated Glenn HT treatment were reconstructed using a Duke reference model, no meaningful results were obtained (Figure 12). Matching the impedances from 16 contacts and 192 voltage measurements resulted in overfitting or extreme conductivity variations that rely on compensation to achieve accurate impedance matches. Thus, reconstruction from eight electrodes was performed. While this produced inferior results when using a subject-specific reference model (lower reconstruction resolution and accuracy), it considerably improved reconstruction using a nonpersonalized reference (Figure 12). Further improvements were obtained when (i) rescaling voltages based on the measurable preheating voltages to compensate for anatomical differences (see Figure 13), (ii) constraining conductivity changes to be positive, and (iii) smoothing the temperature distribution based on the characteristic thermal length (∼1 cm). The latter two improvements are not specific to using personalized reference models.

#### 3.3.2. Temperature and Perfusion Mapping

**Figure 14:**
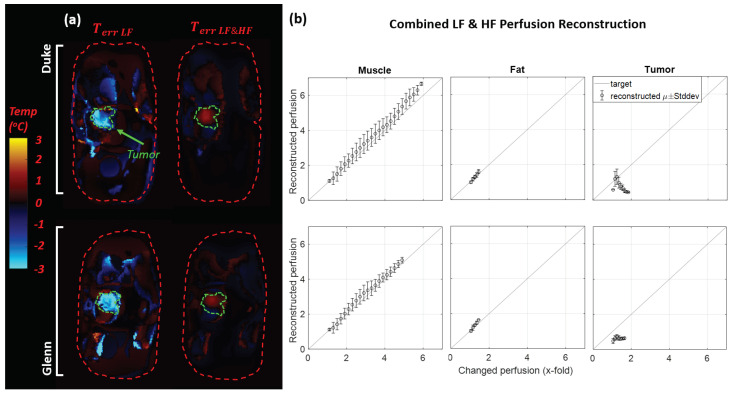
Reconstruction results from multifrequency EIT on Duke and Glenn: (**a**) cross-sectional view of the temperature error distribution; (**b**) reconstructed perfusion versus underlying perfusion plotted separately for muscle, fat, and tumor tissues.

Figure 14 illustrates the perfusion and temperature increase maps obtained using the multifrequency approach from Section 2.4.1 (shown for the Duke case, using the optimistic perfusion model as the reconstruction reference; see Section 2.4). The two-frequency reconstruction resulted in a superior reconstruction of the temperature maps (error < 2 °C), when compared to the single frequency one. The perfusion reconstruction showed important deviations in the tumor. The superiority of the temperature mapping over the perfusion mapping could be related to the differing conductivity reconstruction error magnitudes at the two frequencies. Equation (13) adds different weights to the reconstructions at the two frequencies, such that the two errors are compensated when computing the temperature map, while Equation (14) subtracts the two without weighting, such that part of the conductivity reconstruction error remains and affects the perfusion mapping.

#### 3.3.3. Summary of Temperature Reconstruction Accuracy

Table 3 summarizes the impact of the reconstruction approach and scenario on reconstruction accuracy. It illustrates the improvements relative to the prior state-of-the-art (baseline) afforded by the newly introduced iterative approach, adaptive prior regions, and tissue-dependent penalties. The mean and standard deviation of the reconstruction error in the masked prior region are reported (note that the prior region changes when adaptive prior regions are used). The crucial improvements afforded by the use of planning-based reference models, measurement-based reweighting, and physics-based constraints are not reflected in the table, as the chosen metrics to not allow for a direct comparison across changing anatomies—the relevant information can instead be found in Figure 12. For the generic scenarios, in which the conductivity was changed in a given spherical region, the conductivity reconstruction accuracy was converted to an equivalent temperature accuracy by dividing by *T_c_* = 2%/°C. The limitations of the reconstruction accuracy estimations are discussed in Table 4.

Figure 10 suggests that under ideal conditions and when using personalized reference models, a spatial reconstruction accuracy in the centimeter-range is achievable, which is similar to the inherent thermal length-scale (diffusion related characteristic length of the PBE Green’s function [45]). However, Figure 12 suggests that imperfections, such as the use of nonpersonalized models, reduce the achievable spatial accuracy to multiple centimeters. The ability of detecting highly localized temperature features, e.g., near important cooling arteries, has not been assessed.

## 4. Discussion

In this study, we investigated the potential application of EIT as a low-cost, noninvasive technique for HT treatment monitoring.

For estimation accuracy, if only changes in conductivity associated with $T_{c}$ are considered, a 2% deviation from the actual conductivity leads to 1 °C error in temperature. Due to the presence of perfusion-related conductivity changes, the total conductivity sensitivity to temperature is higher, which can facilitate temperature mapping, if accurate information about the temperature dependence of perfusion is provided. However, the relation between the conductivity error and the temperature or perfusion estimation error becomes more complex. In the absence of well-known temperature–perfusion relationships, distinguishing between perfusion and temperature changes using multifrequency EIT is crucial. To simultaneously map perfusion and temperature changes, the use of more than two measurement frequencies and postprocessing techniques should be further investigated, considering the limited accuracy of conductivity map reconstruction and the limited knowledge about α.

Using a tissue-dependent penalty along with adaptive prior regions are key to achieving conductivity mapping in highly heterogeneous anatomical models. Information from thermal simulations can be used to further improve the accuracy, as shown in the simulated HT treatment scenario. Under ideal conditions, reconstruction in a simulated HT treatment achieves a mean deviation in the order of 1 °C (see Figure 11) in the heated domain. Having a good reference model is important, and a priori personalized models should be used. When using a nonspecific anatomical reference, fewer measurement electrodes should be used and voltages should be rescaled based on preheating measurements. An extreme scenario was investigated to assess local reference model inaccuracies (e.g., passing air bubble). Regarding local reference model inaccuracies (e.g., passing air bubble), an extreme scenario was investigated. We found that using adaptive prior regions in combination with a tissue-dependent penalty successfully enabled reconstruction. Nonablative HT is a relatively long treatment (∼40–60 min) and the heating time constant is in the order of minutes. Therefore, changes during treatment, except for body movements, occur slowly compared with the potential voltage acquisition speed. Continuous monitoring can be combined with continuous prior region adaptation to handle slow tissue environment changes and to adapt the reference model.

Measurement noise is problematic as the number of cells in the model to be reconstructed is large compared to the number of voltage measurements. Figure 9 illustrates the quantifiable impact of SNR on the reconstruction error from 40 dB to 10 dB SNR, while keeping the same reconstruction parameters results in an increase of the reconstruction error by an order of magnitude. The SNR can be improved by averaging multiple acquisitions, and voltage measurements with poor SNR should be detected.

The combination of HT treatment planning with EIT has multiple advantages: Treatment planning typically includes imaging and segmentation for personalized treatment optimization, and a personalized reference model is important for the reconstruction. Electromagnetic and thermal simulations from HT planning can be used in EIT to determine the region where changes in conductivity are expected and which can serve as a prior region. A good reference model for the heated state also facilitates reconstruction (smaller Δσ). In turn, EIT-reconstructed changes in conductivity converted to perfusion/temperature changes can be used for an online adaptation of the treatment plan and of the applied parameters.

For the simulated HT scenarios with the Duke and Glenn ViP models, additional investigations were performed that are not presented in this paper due to space constraints. The following parameters were varied: masking threshold level (37–40 °C), hyperparameter value (0.0001–1), and the number of electrodes (8–32) and electrode rows (2–4). The masking threshold affects the reconstruction of regions with relatively high changes that are left outside the mask, if too high, or the accuracy of high temperature region reconstruction, if too low. The impact of the hyperparameter is dependent on the masking threshold and the mask and penalty values; however, in most of the cases, 0.01 was considered a good choice. Increasing both the number of rows and electrodes did not yield significant improvements compared to two rows with 16 electrodes and resulted in smaller voltage differences between nearby electrodes, making the system more susceptible to noise and measurement uncertainty in practical implementations.

Table 4 lists study limitations that should be considered.

## 5. Conclusions

In this study, we investigated the feasibility of EIT difference imaging for detecting conductivity changes during HT therapy. Realistic scenarios were considered for the practical implementation of EIT in HT monitoring. We implemented an iterative reconstruction in which the reference model was updated for each iteration. The results suggest that a single iteration may be sufficient if there are only small changes.

By using highly heterogeneous anatomical models, we showed that a tissue-dependent penalty parameter improves reconstruction accuracy throughout the modeled volume. We also showed that reconstruction performance has no apparent dependence on the location and extent of the heated region when placing heated spheres with sizes typical of HT heating volumes in relevant torso treatment locations.

Simulated HT treatment with realistic heating patterns revealed large errors in the reconstruction, mainly due to conductivity changes within most of the volume. Using simulated treatment plans as references yields better reconstructions, despite modeling-inherent inaccuracies (e.g., of the tissue parameters). A personalized reference model is thus required; however, a nonspecific reference model can be used if the number of electrodes is reduced and a rescaling of voltages based on preheating measurements is performed.

In view of real-world limitations, we considered the impact of voltage measurement noise and strong localized inaccuracies in the reference model (large air bubble). Both can lead to significant errors, if reconstruction parameters for ideal conditions (no noise, accurate reference model) are used. However, important improvements can be achieved by relaxing reconstruction parameters and introducing prior region adaptation in the reconstruction.

For the successful application of EIT to monitor temperature and perfusion during HT therapy, all factors contributing to the deterioration of the accuracy must be addressed and mitigated. Essentially, accurate reference models (geometry and conductivity) and accurate impedance measurements are required. The results indicate that a temperature estimation accuracy in the order of 1 °C is achievable under the considered conditions and assumptions based on the novel methodologies in this study (iterative reconstruction with adaptive prior regions, planning-based references, measurement-based reweighting, tissue-dependent penalties, and positive heating constraints). The achievable mapping accuracy will depend on how well multifrequency EIT measurements can be leveraged to distinguish direct temperature-related impedance changes from changes caused by perfusion adaptation.

As a next step, experimental realization and validation of the presented approach is required. Initial work could focus on the reconstruction of heating distributions when applying HT ex vivo, where perfusion changes are irrelevant and measurement access is better. Subsequent work would then shift to in vivo situations and rely on strategically placed thermometry catheters, or information from MR thermometry and MR perfusion mapping. Compatibility issues associated with the presence of EIT electrodes during HT application must be considered.

## Figures and Tables

**Table 1:** Size and position of simulated heated region and the added air object for the case of changes outside the focus region. See Figure 2 for the location of the origin (0, 0, 0).

Simulation	P1	P2	P3	P4	P5	Air Object
Center (x, y, z) [mm]	(−90, 20, 15)	(−90, 20, 15)	(−90, 20, 15)	(−40, 50, 15)	(30, 50, 15)	(50, 30, 15)
Diameter [mm]	60	40	80	60	60	50

**Table 2:** Mean and standard deviation of the electrode voltages ($v_{ref}$) and voltage differences at each iteration of reconstruction for two cases (reconstruction using reference conductivity, $\sigma_{ref}$, at 37 °C and $T_{Opt}$).

Electrode Voltages Levels at 1 mA Injection Current				
Simulation	$v_{\mathrm{ref}}$	|Δv| at Iteration		
		1	2	3
Reference 37 °C	5.1 ± 4.8 mV	390 ± 370 μV	42 ± 45 μV	1.7 ± 1.7 μV
Reference $T_{Opt}$	4.7 ± 4.5 mV	30 ± 26 μV	0.9 ± 0.8 μV	0.9 ± 0.8 μV

**Table 3:** Summary of the impact of the reconstruction approach (parameters, iterations, adaptive penalties and prior regions, reference model) and scenario (generic local σ change, detailed treatment scenario, perfusion changes, EIT frequency/frequencies) on the reconstruction accuracy (mean and standard deviation in the prior region). For the realistic HT therapy heating pattern scenarios, the worst case from the investigated Glenn and Duke scenarios is reported. Accuracy of nonpersonalized reconstruction scenarios is not reported here, since the chosen metrics are not applicable.

Temperature Estimation Accuracy	
Condition	Accuracy (Mean/Stddev °C)
Ideal: generic local (spherical) σ change	
1-iter., No Penalty, Hp = 0.01	−3.9/4.9 °C
1-iter., Penalty = 0.001, Hp = 0.01 (*baseline*)	−0.6/1.7 °C
1-iter., Penalty = TiD, Hp = 0.01	−0.6/1.4 °C
3-iter., Penalty = TiD, Hp = 0.01	−0.1/1.1 °C
Nonideal: noise/σ change outside prior region	
3-iter., Penalty = TiD, Hp = 0.01, σ *change outside prior region*	Unusable with Fixed prior region; 0/2.4 °C with Adaptive prior region
3-iter., Penalty = 0.01, Hp = 0.1, SNR = 20 dB	−0.4/3 °C
Hyperthermia treatment scenarios, temperature increase and perfusion changes	
3-iter., Penalty = TiD, HP = 0.01, Ref 37 °C	0.2/1.8 °C (LF) −0.1/1.4 °C (HF)
3-iter., Penalty = TiD, HP = 0.01, *personalized Ref T_Opt_*	−0.3/1.1 °C (LF) −0.1/0.5 °C (HF)
3-iter., Penalty = TiD, HP = 0.01, *Multifrequency*	−0.1/0.3 °C

**Table 4:** List of limitations in this study and their implications.

Limitation	Discussion
Generality	The present study focused on two anatomies, one realistic tumor location in addition to a few spherical heating cases, a fixed exposure element type and placement, and stable EIT electrode placement. While the reconstruction parameters were not particularly tweaked to obtain the presented results, it is important to investigate whether these parameters are indeed generalizable.
Anatomical model accuracy	Personalized anatomical model generation—or intersubject variability, if presegmented models are used—affect the simulation fidelity and are likely to be one of the main sources of reconstruction errors.
Constant $T_{c}$	A constant $T_{c}$ = 2%/°C was used in this study. However, reported values in tissue vary between 0.6–2.1%/°C; refs. [14,46] with a typical value around 2.0%/°C. Although it is unclear how much is measurement-accuracy-related, large intertissue or intersubject variability affects the reconstruction.
Frequency and temperature dependence of conductivity	There is a high degree of uncertainty associated with the temperature dependence of perfusion and electric conductivity. However, since this study focused on conductivity change reconstruction, this uncertainty does not affect our conclusions. If multifrequency EIT can separate direct temperature effects from perfusion-related ones, the uncertainty is reduced to that of $T_{c}$.
Inaccurate conductivity values	The reference model and the model to be reconstructed use tissue properties from a tissue properties database; however, uncertainty and variability associated with these properties affect the achievable reconstruction accuracy. EIT prior to therapy application can help obtain more accurate property maps.
Nonlocal changes of perfusion and local vascular cooling	Although we assumed that increased blood flow circulation occurred in the heated region, nonlocal effects, such as whole-body thermoregulation, convective transport by medium-sized blood vessels, and the stealing effects or blood-flow reduction in a tissue resulting from an increase in neighboring tissue were not considered. Additionally, the localized cooling by sufficiently large blood vessels is not considered by the employed PBE, which assumes distributed perfusion.
Fixed body core temperature	In our simulations, we assumed that body-core temperature was constant. However, the high energy delivery during HT therapy can result in a body-core temperature increase which affects overall tissue temperature.
Electrode modeling and positioning	We modeled point electrodes with precisely known locations. The impact of inaccurate electrode positioning and compensation methods have already been studied [47,48,49]. We assume that accurate placement of electrodes can be assured during treatment. Replacing the point electrodes with extended electrodes will affect the current density in the vicinity of the electrode, and thus the reconstruction sensitivity in that region; this is easily handled and not the subject of this study, which focused on EIT for deep heating monitoring.
Fixed patient geometry	We assumed that the patient geometry did not change between the treatment model creation and the treatment administration. Precise and reproducible patient positioning is required in the clinic, and it is already a requirement for high-quality HT treatment administration. Changes in the internal organ geometry have been investigated in this study, and are handled using adaptive prior regions. Nevertheless, large changes were shown to deteriorate the reconstruction accuracy.
Reconstruction parameter choice	Further investigations are necessary to determine if the reconstruction parameters identified in this study also provide the best reconstruction results across other scenarios.

## Data Availability

No measurement datasets were generated or used in this study. The generated simulations are available on request from the corresponding author. The data cannot be shared publicly due to the licensed anatomical models involved.
